# Estimated Levels of Gluten Incidentally Present in a Canadian Gluten-Free Diet

**DOI:** 10.3390/nu6020881

**Published:** 2014-02-21

**Authors:** Sébastien La Vieille, Sheila Dubois, Stephen Hayward, Terence B. Koerner

**Affiliations:** 1 Director General’s Office, Food Directorate, Health Canada, 251 Sir Frederick Banting Driveway, Ottawa, ON K1A 0K9, Canada; 2 Bureau of Policy, Intergovernmental and International Affairs, Food Directorate, Health Canada, Ottawa, ON K1A 0K9, Canada; E-Mail: sheila.dubois@hc-sc.gc.ca; 3 Bureau of Food Surveillance and Science Integration, Food Directorate, Health Canada, Ottawa, ON K1A 0K9, Canada; E-Mail: stephen.hayward@hc-sc.gc.ca; 4 Bureau of Chemical Safety, Food Directorate, Health Canada, Ottawa, ON K1A 0K9, Canada; E-Mail: terry.koerner@hc-sc.gc.ca

**Keywords:** celiac disease, gluten-free diet, food consumption, grain-containing foods, naturally gluten-free ingredients

## Abstract

Avoiding exposure to gluten is currently the only effective treatment for celiac disease. However, the evidence suggests that for most affected individuals, exposure to less than 10 mg/day is unlikely to cause histological changes to the intestinal mucosa. The daily diet of people with celiac disease does not rely solely on gluten-free pre-packaged foods, but also on naturally gluten-free grains (e.g., rice, buckwheat, ...) and foods with grain-derived ingredients (*i.e.*, flour and starches) used for cooking and baking at home. The objective of this study was to estimate the level of incidental gluten potentially present in gluten-free diets from a Canadian perspective. We have conducted gluten exposure estimations from grain-containing foods and foods with grain-derived ingredients, taking into consideration the various rates of food consumption by different sex and age groups. These estimates have concluded that if gluten was present at levels not exceeding 20 ppm, exposure to gluten would remain below 10 mg per day for all age groups studied. However, in reality the level of gluten found in naturally gluten-free ingredients is not static and there may be some concerns related to the flours made from naturally gluten-free cereal grains. It was found that those containing a higher level of fiber and that are frequently used to prepare daily foods by individuals with celiac disease could be a concern. For this category of products, only the flours and starches labelled “gluten-free” should be used for home-made preparations.

## 1. Introduction

Celiac disease (CD) is a chronic small intestinal immune-mediated enteropathy, observed in genetically susceptible individuals in response to exposure to dietary gluten [1]. Gluten is the main structural protein complex of wheat with equivalent proteins found in other cereals including rye and barley. The gluten toxic protein fractions for people with CD include prolamins and glutenins [2] but the alcohol-soluble fractions (prolamins) of wheat (gliadin), rye (secalin) and barley (hordein) are considered to be the protein constituents of most concern to celiac individuals [3]. The word “gluten” is often considered as a generic name given to storage proteins in wheat, barley, rye and other closely related cereal grains. Thus, it is the gluten in wheat flour that binds and gives structure to bread, baked goods and other foods making it widely used in the production of many processed and packaged foods.

Presently, the only treatment for celiac disease is a strict exclusion of gluten sources including wheat, rye, barley and their hybridized forms from the diet [4,5]. However, a very low amount appears to be tolerated by most of celiac individuals. The available scientific literature addressing safe levels of gluten intake for individuals who suffer from celiac disease essentially relies on either the analysis of results of intestinal biopsies carried out on these individuals or on strictly clinical criteria [6,7]. Although there is no evidence to suggest a single definitive threshold for a tolerable level of gluten intake for those with CD, there is evidence to suggest that, for most affected individuals, exposure to less than 10 mg/day is unlikely to cause histological changes to the intestinal mucosa while exposure to 50 mg/day is likely to do so [8,9].

Typically, the diet that is consumed by individuals who suffer from celiac disease is based on a combination of: (a) foods that naturally contain no gluten and are unlikely to have been contaminated with gluten (meat, fish, milk, eggs, *etc.*); and (b) specially formulated substitute gluten-free grain-containing products (labelled “gluten-free”). However, because naturally gluten-free (GF) grains can become contaminated with gluten-containing grains, there may be some concern about the safety of individuals with CD using naturally gluten-free flours and starches made from cereal grains (e.g., corn), legumes (e.g., peas, beans) or other plants to prepare grain-based foods such as bread and baked goods at home.

The potential for gluten to be present in grains that do not naturally contain gluten is related to the way that grains are grown, harvested and transported. This unintended presence of gluten is to be expected in the context of current industry practices, and is allowed in Canada under the Canadian Grain Standards. There are processes in place for cleaning grains but these can only reduce, and not completely eliminate, the presence of gluten in flours and starches made from cereal plants that do not naturally contain gluten.

The objective of this study was to estimate the level of incidental gluten potentially present in GF diets consumed in Canada, including its potential variability across factors such as presence/absence of a GF package label and varying levels of fiber content in GF grain-based ingredients.

## 2. Materials and Methods

### 2.1. Dietary Intake Data

The dietary intake data used in this study were collected as part of “The Canadian Community Health Survey—Cycle 2.2 on Nutrition, Statistics Canada, 2004” (CCHS 2.2) [10], a survey of the general Canadian population. These data were used because there are no representative data available on food intake by Canadians with celiac disease.

The CCHS 2.2 survey included 35,107 individuals of all ages living in private dwellings in the 10 Canadian provinces and had a response rate of 76.5% [10]. All survey participants provided one 24-h dietary recall; a representative subsample of 10,786 provided a second 24-h dietary recall, 3–10 days later.

A 24-h dietary recall is a retrospective method of dietary assessment in which individuals are interviewed about their food and beverage consumption during the preceding 24 h. A high response rate and an ability to obtain detailed information about food intake (e.g., quantities, cooking methods) are characteristics of this dietary assessment method. While 24-h dietary recalls are believed to influence actual food consumption less than 24-h food records [11], recalls are limited by respondents’ memory about their food consumption.

While 24-h recalls are often used in population surveys such as CCHS 2.2 (the source of dietary intake data used in the present work), single 24-h recalls do not provide good estimates of the usual intake of individuals. This is because there is a large amount of variability in what individuals eat from one day to the next [12].

While the usual intake of individuals cannot be estimated using data from 24-recalls, the distribution of usual intakes of a population can be estimated if a second 24-h recall, for a day that is non-consecutive to the day of the first recall, is obtained for a subset of survey participants. Statistical modelling can then be used to analytically estimate and remove the with-individual, day-to-day variation in dietary intake present in 24-h recall data, leaving the distribution of the between-person variability which is representative of the usual intake distribution of a population [13]. It is this latter population distribution that is of interest in many food policy and food regulatory discussions regarding intakes of dietary components (for example gluten) in targeted populations or sub-populations [14].

### 2.2. Estimated Consumption of Grain-Containing Foods

Consumption of grain-containing foods by participants in CCHS 2.2 was estimated using a three-step procedure.

#### 2.2.1. Step 1: Kinds and Amounts of Grain-Containing Foods Consumed

The Nutrition Survey System (NSS), which was used in the analysis of the 24-h recall data from CCHS 2.2 [15], was used to generate a list of all reported foods that included cereal grains, either as a major ingredient (for example, bread, pasta, breakfast cereals) or as a minor ingredient (for example, puddings, sauces, stews). Each time one of these foods appeared in the first 24-h recall provided by survey participants, the following information was extracted: NSS food code; weight of the food consumed; whether this weight was “as-consumed”.

#### 2.2.2. Step 2: As-purchased Weights of Grain-Containing Foods Consumed

Because section B.24.018 of Canada’s *Food and Drug Regulations* [16] applies to GF foods as sold/purchased, food intakes reported in as-consumed weights were converted to as-purchased weights, as follows: As-consumed weights of simple grain-containing foods, for example cream-of-rice, were converted to as-purchased weights using NSS information on the relative water content of the cooked and uncooked versions. To convert as-consumed weights of mixed dishes that had been prepared at home (for example lasagna) to as-purchased weights of their grain-containing ingredients, information from the recipe component of NSS was used first to calculate the as-consumed weight of each grain-containing ingredient (for example, the weight of the cooked lasagna noodles). Next, the cooked weight of each of these ingredients was converted to an as-purchased weight using NSS information on relative water content of cooked and uncooked versions of the food. As-consumed weights of grain-containing mixed dishes that had been purchased ready-to-eat were not adjusted.

#### 2.2.3. Step 3: Distributions of 24-h and Usual Consumption of Grain-Containing Foods and Ingredients

The one day (24-h) intake of as-purchased weight of grain-containing foods and ingredients by each survey participant was calculated by adding together the as-purchased weights of all grain-containing foods and ingredients consumed on the day of the first 24-h recall provided. Using CCHS 2.2 sampling weights, the 3rd, 5th, 10th, 50th, 90th, 95th and 97th percentiles of the distribution of such consumption were then computed for all survey participants (overall) and for each of 14 age–sex groups: 1–3, 4–8; males and females separately for 9–13, 14–18, 19–30, 31–50 years, 51–70, 71 or above.

Distributions of the usual intakes of grain-containing foods and ingredients (in as-purchased weights) were computed next. The difference in the amount of grain-containing foods consumed between the first and second (repeat) 24-h recalls provided by a subset of survey participants was used to determine the within-individual (day-to-day) variability. The statistical software SIDE was used to adjust the intakes so as to remove the within-individual (day-to-day) variability in the consumption of grain-containing foods) [13]. The resultant distributions reflect the usual consumption of grain-containing foods, for the total survey group and for the 14 age-sex groups.

#### 2.2.4. Estimated Levels of Gluten Exposure

Levels of gluten exposure associated with one-day and usual consumption of GF grain-containing foods were estimated under six exposure scenarios (Table 1). Scenarios 1 and 2 assumed four hypothetical levels of gluten contamination (5 ppm, 10 ppm, 20 ppm or 50 ppm) of as-purchased product. In Scenario 1 all GF substitute foods were purchased ready-to-eat. In Scenario 2, GF cold breakfast cereals and salty snacks were purchased ready-to-eat, GF hot breakfast cereals and pasta were purchased ready-to-cook and all other GF grain-containing foods were prepared at home using pre-packaged GF flours and starches that did NOT have precautionary labelling statements (*i.e.*, that did not have a “may contain” statement). Only pre-packaged GF flours and starches were included in this study; those available for purchase from bulk containers were excluded.

Scenarios 3 to 6 were based on a combination of: (a) GF foods such as bread, baked goods, sauces and puddings which were assumed to have been made at home using naturally GF flours and starches; and (b) an assumed gluten contamination level of 20 ppm (as purchased) in GF foods that cannot readily be made at home (e.g., hot and cold cereal, pasta, salty snacks). For these scenarios statistical modelling (Monte Carlo simulations) [17] was used to generate exposure estimates using the combination of data on: (a) intakes of grain-containing foods reported by participants in CCHS 2.2; (b) information on different combinations of GF flours and starches that can be used to replace wheat flour in GF recipes; (c) factors for converting between the weight of wheat flour and weights of different GF flours and starches used as substitute ingredients; (d) levels of incidental gluten contamination in samples of naturally GF flours and starches purchased in Canada, and analyzed by our team [18].

Scenarios 3 and 5 were based on traditionally used lower fiber GF flours and starches (for example rice flour, tapioca flour). In Scenario 3 packages were labelled as GF; in Scenario 5 they were not labelled as GF. Scenarios 4 and 6 were based on higher fiber GF flours and starches (for example bean flours). In Scenario 4 packages were labelled as GF, in Scenario 6 they were not labelled as GF and both scenarios were based on traditionally used higher fiber GF flours and starches. These latter two scenarios were included because there has been some concern that individuals with CD consuming a GF diet tend to have relatively low levels of fiber intake [19,20]; use of higher-fiber gluten-free flours and starches in baking and cooking is one way to increase fiber intake. For all four of these scenarios, if a package of naturally GF flour or starch did not have a GF label, it was assumed that measures had not been taken to prevent cross-contamination with gluten-containing grains during production, processing, transportation and/or packaging.

The modeling assumptions described above are summarized in Table 2, which also summarizes information on characteristics of food consumption (e.g., one day consumption *versus* usual consumption; consumption by average eaters versus heavy eaters) taken into consideration during statistical modelling.

The specific GF flours and starches for which gluten levels were quantified analytically [18] were chosen to represent a reasonably complete selection of the pre-packaged GF flours and starches readily available to Canadian consumers. Samples were purchased from a variety of grocery and speciality food stores in eight Canadian cities, as well as ordered via the internet. An effort was made to obtain: (a) GF flours and starches with a range of fiber content; as well as (b) packages that were, and packages that were not, labelled GF. All purchases were made via third-parties; product manufacturers were not informed that the purchases were for research purposes. Gluten levels were determined analytically using the RIDASCREEN^®^ R-7001 gliadin ELISA (R-Biopharm Inc., Washington, MO, USA), an assay that has a limit of detection of 3 ppm and a quantification range of 5–80 ppm. Further methodological information is provided in Koerner *et al.* 2013 [18].

**Table 1:** Exposure scenarios.

Scenarios	Product Types	Type of GF Flours and/or Starches	Contamination Levels
1	Bread, muffins, cakes, cookies, cold cereals, salty snacks, puddings, sauces, soups, *etc.*, purchased ready-to-eat; pasta and hot cereal purchased ready-to-cook.	Not specified	5 ppm, 10 ppm, 20 ppm, or 50 ppm as-purchased
2	Bread, muffins, cakes, cookies, puddings, sauces and soups made at home. Cold cereal and salty snacks purchased ready-to-eat; pasta and hot cereals purchased ready-to-cook.	Not specified	5 ppm, 10 ppm, 20 ppm or 50 ppm, as-purchased
3	Bread, muffins, cakes, cookies, puddings, sauces and soups made at home with flours and starches traditionally used in GF cooking and baking.	Flours and starches traditionally used in GF cooking, labelled GF ^a,b^	As determined using RIDASCREEN^®^ R-7001 gliadin ELISA ^c^ (R-Biopharm Inc., Washington, MO, USA) ^d^
	Cold cereal and salty snacks purchased ready-to-eat; pasta and hot cereals purchased ready-to-cook.	Not specified	20 ppm as-purchased
4	Bread, muffins, cakes, cookies, puddings, sauces, soups made at home with flours and starches used in higher-fiber GF cooking and baking.	Flours and starches (>4 g/100 g flour) ^e^ used in higher-fiber GF cooking, labelled GF ^f^	As determined using RIDASCREEN^®^ R-7001 gliadin ELISA
	Cold cereal and salty snacks purchased ready-to-eat; pasta and hot cereals purchased ready-to-cook.	Not specified	20 ppm as-purchased
5	Bread, muffins, cakes, cookies, puddings, sauces and soups made at home using traditional GF flours and starches.	Flours and starches traditionally used in GF cooking, NOT labelled GF	As determined using method RIDASCREEN^®^ R-7001 gliadin ELISA
	Cold cereal and salty snacks purchased ready-to-eat; pasta and hot cereals purchased ready-to-cook).	Not specified	20 ppm as-purchased
6	Bread, muffins, cakes, cookies, puddings, sauces, soups made at home with flours and starches used in higher-fiber GF cooking and baking.	Flours and starches used in higher-fiber GF cooking, NOT labelled GF	As determined using the RIDASCREEN ^®^ R-7001 gliadin ELISA
	Cold cereal and salty snacks purchased ready-to-eat; pasta and hot cereals purchased ready-to-cook).	Not specified	20 ppm as-purchased

^a^: Tapioca flour; yellow and white corn flour; buckwheat flour; quinoa flour; soy flour; potato flour; sorghum flour; white, brown and glutinous (sweet) rice flour; corn starch; potato starch; cornmeal; arrowroot; ^b^: From a popular Canadian cookbook used by Canadian dietitians knowledgeable about celiac disease and a gluten-free diet (Shelley Case, 2008. Gluten-Free Diet: A Comprehensive Resource Guide) and the Canadian Celiac Association web site [21]; ^c^: Enzyme-Linked Immuno-Sorbent Assay; ^d^: See Koerner *et al*., 2013 [18]; ^e^: Fava bean flour, garfava flour, chickpea (garbanzo) flour, peanut flour; almond flour, chestnut flour, hazelnut flour, pecan flour; amaranth flour, millet flour and flax flour; ^f^: For GF recipes using higher fiber ingredients, supplemental internet searches were conducted.

**Table 2:** Characteristics across which variability in levels of intake of incidental gluten was estimated in Scenarios 1–6.

Categories	Characteristics	Scenarios					
		1	2	3	4	5	6
Food consumption	Food consumption on a single day (24-h period)	✓	✓	✓	✓	✓	✓
	Usual food consumption	✓	✓	✓	✓	✓	✓
	Food consumption across specified age and sex groups	✓	✓	✓	✓	✓	✓
	Average eaters (50th) *versus* heavy eaters (97th)	✓	✓	✓	✓	✓	✓
Food preparation	All grain-containing foods purchased ready-to-eat	✓	✗	✗	✗	✗	✗
	Most grain-containing foods prepared at home	✗	✓	✓	✓	✓	✓
Gluten contamination ^a^	Hypothetical levels	✓	✓	✓	✓	✓	✓
	Determined analytically in representative GF flours/starches	✗	✗	✓	✓	✓	✓
GF Labelling	Packages labelled as GF	✗	✗	✓	✓	✗	✗
	Packages NOT labelled as GF	✗	✗	✗	✗	✓	✓
Fiber	Lower-fiber GF flours/starches ^b^	✗	✗	✓	✗	✓	✗
	Higher-fiber GF flours/starches ^c^	✗	✗	✗	✓	✗	✓

^a^: Scenarios 3–6 used the combination of analytically determined levels used for GF foods readily prepared at home (e.g., muffins) and a hypothetical level of 20 ppm used for GF foods less readily prepared at home (e.g., crackers); ^b^: Flours/starches traditionally used in GF cooking and baking (e.g., rice flour); ^c^: Flours/starches sometimes used to increase fiber intake (e.g., bean flour).

In preparation for the statistical modelling for Scenarios 3 to 6, commonly used home recipes for GF substitute foods such as bread, muffins, cakes, cookies and pies were identified, as were the GF flours and starches commonly recommended for thickening sauces, gravies and stews. Information was compiled on specific types and volumes of the different GF flours and starches used in each identified recipe. Volume/weight conversion factors were used for each GF flour and starch.

During the statistical modelling, whenever a grain-containing food for which a GF substitute could be readily prepared at home appeared in a 24-h recall, a recipe was selected at random from the appropriate subset of assembled recipes (for example, the assembled set of GF cookie recipes). The different GF flours and/or starches used in the recipe and their relative proportions were noted. A gluten level for each flour/starch was selected at random from among the measured levels. The gluten level in each GF grain-containing substitute food was then estimated by summing the measured level of gluten determined for each GF flour and starch included in the selected recipe, adjusting for differences between the weight of each GF flour or starch and the weight of wheat flour which was assumed to be the primary type of flour present in grain-based foods consumed by participants in CCHS 2.2.

## 3. Results

Estimated intakes of grain-containing foods (g/day) by average eaters (50th percentile) and by heavy eaters (97th centile) in the CCHS 2.2 survey are presented in Table 3. Estimates are presented for both single day intakes and for usual intakes, as well as for both “as-consumed” weights and “as-purchased” weights. “As-purchased” weights are reported because section B.24.018 of Canada’s *Food and Drug Regulations* applies to GF foods as sold/purchased.

**Table 3:** Estimated intake (g) of grain-containing foods among CCHS 2.2 participants.

Categories	24-h Intake		Usual Intake	
	50th	97th	50th	97th
Grain-containing foods, as-consumed weight (g)	154.0	618.5	189.2	391.0
Grain-containing foods, as-purchased weight (g)	133.5	433.3	151.4	299.6

Estimated levels of gluten exposure under Scenarios 1 and 2 (hypothetical gluten contamination levels of 5, 10, 20 and 50 ppm) among all survey respondents are presented in Table 4. Estimates are presented separately for a single 24-h period and for usual intake; for average eaters (50th percentile) and for heavy eaters (97th percentile). Looking at the results for Scenario 1, hypothetical contamination levels of 5 ppm and 10 ppm in all ready-to-eat GF grain-containing foods was associated with an estimated gluten exposure levels below 4 mg/day, among both average eaters (50th percentile) and heavy eaters (97th percentile). At a hypothetical contamination level of 50 ppm the estimated total daily intake at the 97th percentile is 19.31 mg/day for one-day and 14.98 mg/day for usual intake, both higher than the 10 mg/day tolerated by most individuals with celiac disease. A hypothetical contamination rate of 20 ppm was associated with one-day and usual gluten exposure estimates for heavy eaters (97th percentile) below 10 mg/day (7.72 mg/day and 5.64 mg/day respectively).

**Table 4:** Estimated percentiles of gluten exposure (mg/day) assuming hypothetical levels of gluten contamination of 5, 10, 20 or 50 ppm in as-purchased products.

Gluten Levels	Scenario 1 Food Purchased Ready-to-Eat				Scenario 2 Ingredients Purchased for Home Preparation			
	24-h Intake		Usual Intake		24-h Intake		Usual Intake	
	50th	97th	50th	97th	50th	97th	50th	97th
5 ppm	0.63	1.93	0.76	1.50	0.48	1.55	0.55	1.08
10 ppm	6	3.86	1.51	3.00	0.97	3.11	1.09	2.16
20 ppm	2.54	7.72	3.03	5.64	1.93	6.21	2.19	4.32
50 ppm	6.34	19.31	7.57	14.98	4.84	15.53	5.47	10.80

Table 5 provides separate gluten exposure estimates for 14 age-sex groups under the 20 ppm hypothetical level of gluten contamination used in Scenarios 1 and 2. While exposure estimates vary across these population sub-groups it can be seen that the highest estimated exposure levels among heavy eaters (97th percentile) are for males 19–30 years of age and males 14–18 years of age. While estimated exposure for these two sub-groups is slightly over 10 mg/day when a single day’s food consumption is considered (10.31 mg/day and 10.12 mg/day respectively), it is below 10 mg/day when usual dietary intakes are considered (8.45 mg/day and 4.92 mg/day respectively). Exposure estimates are generally lower for Scenario 2 than for Scenario 1, with 97th percentile values for Scenario 2 of 8.45 mg/day (one day) and 4.92 mg/day (usual) for males 19–30 years of age and values of 8.33 mg/day and 5.75 mg/day respectively for males 14–18.

**Table 5:** Estimated percentiles of gluten exposure (mg/day) in different age-sex groups assuming hypothetical levels of gluten contamination of 20 ppm.

Age-Sex Groups	Scenario 1 Food purchased Ready-to-Eat				Scenario 2 Ingredients purchased for Home Preparation			
	24-h Intake		Usual Intake		24-h Intake		Usual Intake	
	50th	97th	50th	97th	50th	97th	50th	97th
1–3 years	1.59	4.60	1.73	3.65	1.25	3.89	1.35	2.91
4–8 years	2.71	6.90	2.94	4.67	2.06	5.27	2.28	3.66
M: 9–13 years	3.30	8.48	3.75	5.66	2.52	7.18	2.89	4.31
F: 9–13 years	2.88	6.86	3.19	5.06	2.16	5.78	2.46	3.90
M: 14–18 years	3.67	10.12	4.13	7.12	2.78	8.33	3.22	5.75
F : 14–18 years	2.75	7.58	2.95	5.33	2.07	6.13	2.30	4.25
M: 19–30 years	3.35	10.31	3.71	6.30	2.55	8.45	2.96	4.92
F: 19–30 years	2.34	6.80	2.49	4.28	1.82	5.66	2.00	3.56
M: 31–50 years	2.89	8.95	3.31	6.47	2.21	7.15	2.57	4.56
F: 31–50 years	2.23	6.33	2.43	4.14	1.71	4.84	1.90	3.48
M: 51–70 years	2.70	7.10	2.86	5.36	2.06	5.92	2.19	4.22
F: 51–70 years	2.06	5.88	2.22	4.01	1.55	5.02	1.74	3.35
M: ≥71 years	2.51	6.75	2.72	4.81	1.91	5.26	2.08	3.80
F: ≥71 years	2.03	4.83	2.16	3.83	1.53	3.97	1.65	2.97
Overall	2.54	7.72	3.03	5.64	1.93	6.21	2.19	4.32

M: Male; F: Female.

Table 6 presents results for the two lower fiber scenarios (Scenarios 3 and 5) in which many grain-based foods are prepared at home with packages of traditionally used GF flours and starches (for example, rice flour, corn starch, potato starch). In Scenario 3 all packages of flour/starch had GF labels. As can be seen on the left side of Table 6, estimated gluten intake is consistently below 10 mg/day, for a single day’s intake and for usual intake, for average eaters (50th centile) and for heavy eaters (97th centile), overall and in each of the 14 age-sex groups for which intake levels were estimated.

Scenario 5 was similar to Scenario 3 except that in Scenario 5 none of the packages of GF flour/starch had GF labels. Results for Scenario 5 are similar to those for Scenario 3. As can be seen on the right side of Table 6, values for the (97th percentile for a single day ranged from a low of 2.81 mg/day for females 71 years of age or older to a high of 7.03 mg/day for males 19–30 years of age. Corresponding values for usual intake were somewhat lower (from 2.05 for females 71 years of age or older to 4.21 for males 14–18 years of age).

Table 7 presents results for the two higher fiber scenarios (Scenarios 4 and 6) in which many grain-based foods are prepared at home using packages of GF flours and starches that can be used to increase the fiber content of a GF diet (for example, bean flour). In Scenario 4 all packages had GF labels. As can be seen on the left side of Table 7, estimated gluten intake is below 10 mg/day, for both a single day’s intake and for usual intake, for average eaters (50th percentile) and heavy eaters (97th percentile), overall and in each of the 14 age-sex groups for which intake levels were estimated.

**Table 6:** Estimated percentiles of gluten exposure (mg/day) in different age-sex groups when many grain-containing foods are prepared at home using traditional lower fiber GF flours and starches.

Age-Sex Groups	Scenario 3 Lower Fiber GF Flours and Starches Labelled GF				Scenario 5 Lower Fiber GF Flours and Starches NOT Labelled GF			
	24-h Intake		Usual Intake		24-h Intake		Usual Intake	
	50th	97th	50th	97th	50th	97th	50th	97th
1–3 years	0.52	2.94	0.70	1.91	0.59	3.13	0.80	2.39
4–8 years	0.86	4.35	1.20	2.44	0.98	4.52	1.39	2.88
M: 9–13 years	0.79	6.04	1.29	2.95	1.01	6.09	1.52	3.19
F: 9–13 years	0.74	5.06	1.17	2.43	0.92	5.06	1.38	2.70
M: 14–18 years	0.83	6.55	1.44	3.69	1.04	6.65	1.70	4.21
F: 14–18 years	0.54	4.67	0.86	2.90	0.73	4.83	1.11	3.25
M: 19–30 years	0.18	7.01	1.05	5.65	0.61	7.03	1.24	4.10
F: 19–30 years	0.46	4.26	0.78	2.16	0.60	4.48	0.96	2.65
M: 31–50 years	0.21	5.90	0.97	2.92	0.57	5.99	1.18	2.58
F: 31–50 years	0.29	3.71	0.63	2.33	0.50	3.91	0.82	2.48
M: 51–70 years	0.29	4.22	0.61	2.97	0.56	4.40	0.86	2.80
F: 51–70 years	0.29	3.10	0.53	2.08	0.48	3.40	0.70	2.23
M: ≥71 years	0.53	3.27	0.66	2.55	0.70	3.62	0.87	2.58
F: ≥71 years	0.43	2.43	0.54	1.72	0.56	2.81	0.74	2.05
Overall	0.51	4.54	0.76	2.94	0.68	4.74	1.00	3.19

**Table 7:** Estimated percentiles of gluten exposure (mg/day) in different age-sex groups when many grain-containing foods are prepared at home using higher fiber GF flours and starches.

Age-Sex Groups	Scenario 4 Higher Fiber GF Flours and Starches Labelled GF				Scenario 6 Higher Fiber GF Flours and Starches NOT Labelled GF			
	24-h Intake		Usual Intake		24-h Intake		Usual Intake	
	50th	97th	50th	97th	50th	97th	50th	97th
1–3 years	0.56	3.02	0.74	1.95	0.96	22.96	2.73	9.44
4–8 years	0.93	4.46	1.25	2.42	1.68	29.89	5.29	12.30
M: 9–13 years	0.92	5.98	1.40	2.59	2.06	41.47	7.44	12.59
F: 9 –13 years	0.86	5.06	1.28	2.12	1.75	33.70	6.23	11.52
M:14–18 years	0.94	6.46	1.53	3.46	2.22	51.50	8.81	17.05
F: 14 –18 years	0.66	5.05	1.03	2.76	1.47	38.91	5.69	13.22
M: 19–30 years	0.47	7.12	1.20	3.85	1.77	49.43	7.58	14.96
F: 19–30 years	0.56	4.72	0.90	2.28	1.24	32.54	4.94	9.77
M: 31–50 years	0.47	6.39	1.12	2.33	1.56	47.42	6.37	17.99
F: 31–50 years	0.44	4.05	0.78	2.10	1.05	32.10	4.61	10.73
M: 51–70 years	0.47	4.79	0.84	2.56	1.42	41.17	6.50	14.19
F: 51–70 years	0.43	3.76	0.70	1.86	1.08	30.16	4.79	10.31
M: ≥71 years	0.65	4.03	0.86	2.27	1.46	34.24	5.89	11.87
F: ≥71 years	0.52	3.12	0.71	1.71	1.13	27.48	4.68	10.52
Overall	0.61	4.87	0.96	2.56	1.42	37.66	5.71	13.65

Scenario 6 was similar to Scenario 4 except that in Scenario 6 none of the packages of GF flour/starch had GF labels. However, results for Scenario 6 are quite different to those for Scenario 4. As can be seen on the right side of Table 7, values for the 97th percentile for a single day ranged from a low of 22.96 mg/day for children 1–3 years of age to a high of 51.50 mg/day for males 19–30 years of age. Corresponding values for usual intake were somewhat lower (from 9.44 mg/day for children 1–3 years of age to 17.99 mg/day for males 31–50 years of age).

## 4. Discussion

For people with celiac disease, the main concern regarding the use of “gluten-free” prepackaged products and the use of naturally gluten-free food is the doubt that these products being “contaminated” with the main sources of gluten, *i.e.*, wheat, rye and barley.

The adoption of a single gluten threshold for gluten contamination at 20 mg/kg (ppm) is suggested by several authors [8,22]. Thus, in Canada, risk evaluators consider that the presence of gluten at levels which do not exceed 20 ppm in products that are labelled “gluten-free” does not pose a risk for the vast majority of individuals who suffer from celiac disease [23]. The choice of the 20 ppm level for the purposes of risk management reflects also an international standard that has been initially established by the Codex Alimentarius Commission based on scientific premises [24]*.* A number of western countries have ruled on this matter, and have implemented the 20 ppm level indicated in the Codex Alimentarius standard. Thus, the measure is effective since January 2012 in the European Union (*Commission Regulation (EC) No 41/2009 of 20 January 2009*) and was announced in August 2013 by the US-Food and Drug Administration.

The 20 ppm level is used to express the concentration of gluten present in a given food, but the measurement of total exposure to gluten each day is what constitutes the threshold in terms of prevention (10 mg/day). Therefore, by applying the 20-ppm level for gluten-free foods, with the understanding that total gluten intake must be limited to no more than 10 mg/day, it would be possible to consume 500 g of gluten-free foods on a daily basis. However, this calculation should take account the exposure to flours made from cereal grains and plants that do not contain gluten naturally and that are frequently used by celiac consumers to prepare daily foods.

Overall, the question is to know whether the limit of 20 ppm is a realistic threshold to protect most of the Canadian people with CD taking account Canadian consumption data including “gluten-free” prepackaged products but also naturally gluten-free food notably flours and starches used at home by celiac consumers for GF cooking and baking.

Our results show that for a hypothetical level of contamination of 50 ppm the estimated usual daily intake at the 97th percentile was 14.98 mg/day in Scenario 1 (ready-to-eat) and 10.80 mg/day in Scenario 2 (mainly prepared at home). Both are above the maximum safe level of 10 mg/day gluten intake for individuals with celiac disease. We conclude that, given the consumption levels of grain-containing foods by Canadian in the general population (levels that may not currently be consumed by Canadians with celiac disease, but could be in the future, to the extent that the perceived quality of GF grain-based products increases and their cost decreases), from a safety perspective this level of gluten contamination is too high for prepackaged GF foods or ingredients labelled as GF and sold in Canada.

For a hypothetical level of contamination of 20 ppm, estimated usual daily intake at the 97th percentile (heavy Canadian consumers) averaged 5.6 mg/day for Scenario 1 (ready-to-eat) and 4.3 mg/day for Scenario 2 (mainly prepared at home) (Table 4). While exposure estimates are fairly similar for each of the hypothetical levels of gluten exposure examined in Scenarios 1 and 2, exposure estimates are consistently lower in Scenario 2. This can be explained by the fact that the hypothetical levels of gluten contamination were applied (in most cases) to ingredients such as flours and starches in Scenario 2 while they were applied to ready-to-eat foods in Scenario 1. The dry weights of the flours and starches used as ingredients in Scenario 2 are less than the weights of the corresponding ready-to-eat grain-containing foods in Scenario 1, thus exposure estimates are expected to be lower.

While there was variation in intake across the different age and sex groups, the 97th percentile estimates did not exceed 10 mg/day in any group: the highest 97th percentile values were 7.1 mg/day and 6.3 mg/day for males 19–30 years of age and males 14–18 respectively. On the basis of these results we conclude that a maximum of 20 ppm of incidental gluten in pre-packaged GF grain-containing foods and ingredients that are labelled as GF and sold in Canada does not pose a health risk for the majority of Canadians with celiac disease.

In Scenarios 3 to 6, instead of using hypothetical levels of gluten contamination, we used data our team had previously collected on levels of gluten contamination of a range of pre-packaged GF flours and starches sold in Canada [18]. We then estimated dietary exposures using these measured values in GF grain-based foods that can be prepared at home relatively easily (bread, cake, cookies), while continuing to assume that GF grain-based foods that are more difficult to prepare at home (e.g., pasta, crackers) were contaminated at levels of 20 ppm (as-purchased).

Considering results by age-sex group (Table 5) and assuming a gluten contamination level at 20 ppm, the highest estimated exposure levels among heavy eaters (97th percentile) are for males 19–30 years of age and males 14–18 years of age and are slightly over 10 mg/day considering a single day’s food consumption (10.12 mg/day and 10.31 mg/day respectively). With a contamination level at 20 ppm, this category of consumers is susceptible to consume a little bit more than 500 g of prepackaged products per day and with a diagnosis of celiac disease, young male adults should be encouraged to reduce their consumption of food ready-to-eat (*i.e.*, prepackaged food with a “gluten–free” statement). However, considering usual intake measurements which correspond to a more realistic situation than a single day’s food consumption, the highest estimated exposure level is below 10 mg/day for the same categories of consumers (7.1 mg/day and 6.3 mg/day respectively). Moreover, the values of 10.1 mg/day and 10.3 mg/day are very close to 10mg/day observed with 24-h intake assessment. On the basis of these results, we would consider that the limit of 20 ppm defined as a threshold value for celiac disease risk management should not be questioned from a public health point of view.

Lower fiber GF flours and starches traditionally used in GF cooking and baking were used in Scenarios 3 (packages labelled as GF) and 5 (packages NOT labelled as GF, with no precautionary statement, *i.e.*, “may contain”). Gluten exposure at the 97th percentile was only slightly higher in Scenario 5 (3.19 mg/day) than in Scenario 3 (2.94 mg/day). Possible explanations for the similarity of these results include the fact that grains such as rice and corn are generally grown, transported, processed and sometimes packaged in different regions of the world than wheat is. Consequently, the level of cross-contamination is low even with non-GF products. Until this finding of similar levels of gluten contamination in lower-fiber GF flours and starches labeled GF and comparable products not labeled as GF are demonstrated to be a consistent characteristic of lower-fiber GF flours and starches not labeled as GF, we recommend that Canadians with celiac disease continue to use only pre-packaged lower fiber GF flours and starches that are labelled as GF.

Looking at diets with GF grain-containing foods prepared at home using higher GF fiber flours (table 8) and starches with GF labels (Scenario 4), estimated usual intake of gluten at the 97th percentile is well below 10mg/day in all age-sex groups (1.71 to 3.46). In contrast, results of the modeling for Scenario 6 (higher GF fiber flours and starches with no GF label), show that all but two of the 97th percentile values for usual intake by age-sex are greater than 10 mg/day (10.31 to 17.05). While usual intakes among average eaters (50th percentile) were, as expected lower (2.73–7.58), they were also considerably higher than the corresponding 50th percentile estimates for lower fiber GF flours and starches not labeled as GF (0.74–1.70), suggesting that concern should not be restricted to individuals with the highest consumption of GF grain-containing foods. We recommend that individuals with celiac disease who wish to increase their fiber intake by using higher-fiber flours and starches purchase only packages that are labeled as GF.

In preliminary results of this study, Koerner *et al*. [18] have shown that traditional ingredients (considered here as lower fiber content) have a lower contamination compared with those with higher fiber content such as soy, millet and buckwheat. As it was initially suggested in Koerner’s study, our exposure scenarios confirm that among the naturally gluten-free flours and starches tested that do not contain a gluten-free label, the higher fiber ingredients would constitute the greatest probability of being contaminated with gluten above 20 mg/kg and should be avoid by celiac consumers.

## 5. Conclusions

Despite some limitations, based on exposure estimations to gluten in usual intake, and taking into consideration: (a) the various rates of food consumption by different sex and age groups in the Canadian population; and (b) the level of gluten contamination of GF flours and starches available on the Canadian marketplace which were published in a previous study [18], our results have shown that if gluten was present at levels not exceeding 20 ppm, exposure to gluten would remain below 10 mg per day for all age groups studied. Nevertheless, there may be some concerns related to flours made from cereal grains that do not contain gluten naturally especially those containing a high level of fibers and that are frequently used to prepare daily foods by individuals with celiac disease. For this category of products sold in Canada, only the flours and starches labeled “gluten-free” should be used for cooking and baking at home.

- *Flours/starches traditionally used in gluten-free cooking and baking* [18]: Arrow roots, corn starch, potato starch, rice flour—white, rice flour—sweet, flour mix, tapioca starch.
- *Flours/starches sometimes used to increase the fiber content of gluten-free cooking and baking* [18]: Amaranth flour, buckwheat flour, nut flours, corn flour/meal, flaxseed meal, bean flour, pea flour, millet flour, potato flour, quinoa flour, rice flour-brown, sorghum flour, soy flour.
